# Combatting Planktonic and Biofilm Populations of Carbapenem-Resistant *Acinetobacter baumannii* with Polymyxin-Based Combinations

**DOI:** 10.3390/antibiotics11070959

**Published:** 2022-07-16

**Authors:** Marisol Wences, Elliot R. Wolf, Cindy Li, Nidhi Singh, Nene Bah, Xing Tan, Yanqin Huang, Zackery P. Bulman

**Affiliations:** Department of Pharmacy Practice, College of Pharmacy, University of Illinois Chicago, Chicago, IL 60612, USA; mwence2@uic.edu (M.W.); ewolf3@uic.edu (E.R.W.); xli234@uic.edu (C.L.); nidhis04@uic.edu (N.S.); nbah2@uic.edu (N.B.); xintan2391@gmail.com (X.T.); yhuan61@uic.edu (Y.H.)

**Keywords:** *A. baumannii*, carbapenem, biofilm, combinations, polymyxin

## Abstract

Carbapenem-resistant *Acinetobacter baumannii* (CRAB) can cause serious infections that are associated with high mortality rates. During the course of an infection, many CRAB isolates are able to form biofilms, which are recalcitrant to several antibiotics and can be difficult to treat. Polymyxin-based regimens are a first-line treatment option for CRAB infections, but they have not been optimized against both planktonic and biofilm phases of growth. The objective of this study was to identify polymyxin-based combinations that are active against planktonic and biofilm populations of CRAB. Four CRAB isolates (meropenem MICs: 8–256 mg/L) capable of forming biofilms were used in each experiment. The activities of polymyxin B alone and in combination with ampicillin/sulbactam, meropenem, minocycline, and rifampin were assessed using time-kill assays, with the CRAB isolates grown in planktonic and biofilm phases. Viable colony counts were used to detect the bactericidal activity and synergy of the antibiotic combinations. Against the planktonic populations, polymyxin B combined with meropenem, minocycline, ampicillin/sulbactam, and rifampin caused 3.78, −0.15, 4.38, and 3.23 mean log_10_ CFU/mL reductions against all isolates at 24 h, respectively. Polymyxin B combined with meropenem, ampicillin/sulbactam, or rifampin was synergistic against 75–100% (3/4 or 4/4) of CRAB isolates. Against biofilms, polymyxin B combined with meropenem, minocycline, ampicillin/sulbactam, and rifampin caused 1.86, 1.01, 0.66, and 3.55 mean log_10_ CFU/mL reductions against all isolates at 24 h, respectively. Only the combination of polymyxin B and rifampin retained bactericidal activity or synergy against any of the isolates when grown as biofilms (50% of isolates). The combination of polymyxin B and rifampin may be promising for CRAB infections that have planktonic and biofilm populations present.

## 1. Introduction

*Acinetobacter baumannii* is a common cause of ventilator-associated pneumonia and catheter-related infections, largely affecting individuals with prolonged hospital stays and/or immunocompromised health [1]. Many *A. baumannii* are resistant to carbapenems, a last resort class of antibiotics for multidrug-resistant Gram-negative bacteria, leaving few therapeutic options available [2]. The World Health Organization has designated carbapenem-resistant *Acinetobacter baumannii* (CRAB) as a critical priority pathogen due to its association with high rates of morbidity and mortality [3]. The high levels of resistance to carbapenems and other antibiotic classes in CRAB has forced clinicians to resort to last-line treatment options such as the polymyxins (colistin or polymyxin B). Clinical failure rates of up to 79% have been reported for polymyxin monotherapy when used to treat CRAB infections [4,5,6]. The current polymyxin guidelines recommend using a polymyxin in combination with one or more additional active antibiotics when treating a CRAB isolate that is susceptible to at least one non-polymyxin agent [7]. However, the optimal antibiotic to combine with a polymyxin for treatment of CRAB infections remains unclear. The challenge of treating *A. baumannii* is further exacerbated by its ability to form biofilms, which create an added barrier for antimicrobial therapy [8].

*A. baumannii* can form biofilms on abiotic surfaces such as endotracheal tubes during ventilation or indwelling catheters, which contribute to this pathogen’s propensity to cause nosocomial infections [8]. *A. baumannii* can also establish biofilms on biotic surfaces, such as the lungs during pneumonia, by adhering to respiratory epithelial cells [9,10,11]. Once attached to the host’s respiratory epithelium, the bacteria can aggregate to form multicellular clusters of bacteria (biofilms), which act in a coordinated manner, controlled by quorum sensing [12,13]. Biofilms exploit host cells for nutrients and excrete an extracellular polymeric matrix composed of exopolysaccharides, biofilm-associated proteins, and DNA, which can protect the bacterial cells from antibiotics [8,14]. The ability of *A. baumannii* to grow in planktonic and biofilm populations highlights the importance of selecting antimicrobial agents with activity against isolates in both growth phases. Previous studies have primarily assessed the antimicrobial activities of polymyxin-based combinations against planktonic or biofilm populations separately. In this study, we conducted experiments to compare the in vitro activity of polymyxin B-based combinations for planktonic and biofilm phases using four meropenem-resistant (MIC 8–256 mg/L) CRAB isolates with polymyxin B MICs between 0.5 and 1 mg/L.

## 2. Materials and Methods

### 2.1. Bacterial Isolates and Materials

Four clinical CRAB isolates (AR-276, AR-279, AR-285, and AR-313) were used in each experiment [15]. The sequences of these isolates were downloaded from GenBank for analysis. Whole-genome sequencing was previously performed by the Center for Disease Control and Prevention. Multilocus sequence typing (MLST) was performed using PubMLST and the ST was reported using the Pasteur scheme [16]. Genomes were analyzed with the ResFinder 4.1 and CARD databases to identify β-lactamase genes harbored by each isolate [17,18]. These isolates were selected based on their biofilm-forming potential and meropenem MICs (range from 8 to 256 mg/L) (Table 1). Isolates that were meropenem resistant were screened for adequate biofilm growth using a Calgary device, which has a lid with pegs that facilitate biofilm growth, as previously described [19]. Briefly, an initial inoculum of ~10^7^ CFU/mL was added to 96-well flat bottom plates and covered by a lid with pegs. Biofilms were formed on pegs over 20 h in 37 °C while rocking. Pegs were rinsed with normal saline and then the biofilms were transferred to the wells by centrifugation. A baseline (0 h) optical density measurement at 650 nm (OD_650_) using a microtiter plate reader was taken and compared to OD_650_ after 6 h of growth at 37 °C. Adequate biofilm growth for the positive control (ATCC 19606) and clinical isolates was defined as an OD_650_ difference (6 h OD_650_−0 h OD_650_) of ≥0.05. The four selected isolates (AR-276, AR-279, AR-285, and AR-313) that were determined to form biofilms then underwent testing to quantify their biofilm formation potential, as previously described [19,20]. Briefly, the CRAB isolates were grown for 48 h at 37 °C in a Calgary device. Isolates were inoculated at ~10^6^ CFU/mL in Cation-adjusted Mueller Hinton broth (CAMHB) (BD Difco). Mature biofilms were then washed, stained with 0.1% crystal violet for 15 min, and then dried. Dried pegs were then submerged in 33% glacial acetic acid for 1 h, followed by optical density measurement at 570 nm using a plate reader. Experiments were performed in triplicate. The mean OD_570_ values from biofilms (OD_Biofilm_) of each isolate were compared to the optical density cut-off (ODc) from the broth-only control, as described previously [21]. Isolates were categorized as displaying weak (ODc < OD_Biofilm_ ≤ 2 × ODc), moderate (2 × ODc < OD_Biofilm_ ≤ 4 × ODc), or strong (OD_Biofilm_ > 4 × ODc) biofilm-forming capacity.

CAMHB was used as the growth medium for each experiment. Ampicillin (AMP, LOT #089M4784V), minocycline (MIN, LOT #058M4031V), polymyxin B (PMB, LOT #088M4029V), and rifampin (RIF, LOT #MFCD00151389) were obtained from Sigma-Aldrich. Meropenem (MER, LOT #LC42091) and sulbactam (SUL, LOT #02C7M16J) were obtained from AK Scientific. The MICs for all four isolates were determined against the six antibiotics of interest in accordance with the Clinical and Laboratory Standards Institute (CLSI) guidelines [22].

### 2.2. Planktonic Time-Kill Assays

Static time-kill assays against planktonic CRAB isolates were performed as previously described [23,24]. Each experiment was started at an inoculum of ~10^6^ CFU/mL in 20 mL of CAMHB and grown with continuous shaking at 37 °C. CRAB isolates were exposed to 0.25 × MIC, 0.5 × MIC, 1 × MIC, 2 × MIC, and 4 × MIC of AMP/SUL, MER, MIN, PMB, and RIF alone. Combinations (PMB/AMP/SUL, PMB/MER, PMB/MIN, and PMB/RIF) were tested at 0.5 × MIC of each antibiotic. Samples were obtained after 0, 1, 2, 4, 6, 8, and 24 h for viable colony counting. Experiments were performed in duplicate. Bactericidal activity was defined as a ≥3 log_10_ CFU/mL reduction in viable cell count and synergy was defined as a ≥2 log_10_ CFU/mL reduction by the combination compared to the most active agent alone at 24 h.

### 2.3. Biofilm Time-Kill Assays

Biofilm static time-kill assays were performed against each CRAB isolate at an inoculum of ~10^8^ CFU/mL. CRAB biofilms of each isolate were initially formed in 200 μL of CAMHB for 24 h at 37 °C in a 96-well round bottom microtiter plate, as previously described [25]. After 24 h of growth, the existing 200 μL of media was removed from each well. After removal of the broth, antibiotics and fresh CAMHB were loaded into each well. In the growth control arms, CAMHB alone was added to the wells. Antibiotics were assessed alone (AMP/SUL, MER, MIN, PMB, and RIF) and in combinations (PMB/AMP/SUL, PMB/MER, PMB/MIN, and PMB/RIF) against the CRAB biofilms. Since biofilms are more recalcitrant to antibiotics than planktonic cells, only 4 × MIC concentrations were used for drugs alone and in combinations. The first time point (0 h) was obtained immediately after the introduction of the antibiotic regimens and subsequent samples were obtained 2, 4, 6, and 24 h thereafter. At each time point, the biofilms were dislodged from the wells with a 1 μL inoculation loop, serially diluted, and plated for viable colony counting. Experiments were performed in triplicate and the mean viable counts are reported. Bactericidal activity was defined as a ≥3 log_10_ CFU/mL reduction in viable cell count and synergy was defined as a ≥2 log_10_ CFU/mL reduction by the combination compared to the most active agent alone at 24 h [25,26].

## 3. Results

### 3.1. Isolate Characteristics

AR-279, AR-285, and AR-313 were determined to be *A. baumannii* while AR-276 was a member of the *A. baumannii* complex. The AR-276, AR-279, AR-285, and AR-313 CRAB isolates belonged to ST530, ST2, ST79, and ST1, respectively (Table 1). MICs for PMB were below the resistance breakpoint for all isolates and ranged from 8 to 256 mg/L for MER. Each CRAB isolate harbored a unique profile of β-lactamase genes. Several isolates (AR-279, AR-285, and AR-313) harbored a gene that encodes an enzyme known to hydrolyze carbapenems. AR-276, AR-279, and AR313 had moderate biofilm-forming capacity while AR-285 had weak biofilm-forming capacity.

### 3.2. Planktonic Time-Kill Assays

**Figure 1:**
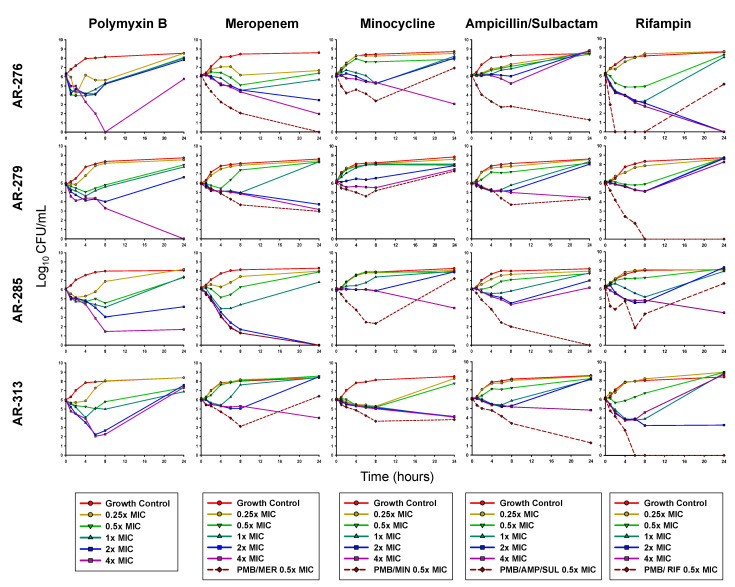
Mean viable bacterial counts of planktonic CRAB in static time-kill experiments. For combinations, each antibiotic was tested at a concentration of 0.5 × MIC. PMB, polymyxin B; MER, meropenem; MIN, minocycline; AMP/SUL, ampicillin/sulbactam; RIF, rifampin.

As monotherapy, 4 × MIC PMB was bactericidal at 24 h against AR-279 and AR-285 (Table 2; Table S1). Against AR-276, 4 × MIC of PMB displayed rapid initial killing, which resulted in undetectable bacterial counts at 8 h, followed by regrowth to near the starting inoculum by 24 h (Figure 1). PMB (2 × and 4 × MIC) was bactericidal against isolate 313 between 6 and 8 h but regrowth to ≥6 log_10_ CFU/mL was observed by 24 h. At 24 h, MER 4 × MIC caused 2.77 and 2.08 log_10_ CFU/mL reductions in AR-279 and AR-313, respectively, and was bactericidal against AR-276 and AR-285. MIN at 4 × MIC caused ~2–3 log_10_ CFU/mL killing against AR-276, AR-285, and AR-313, which was also considered bactericidal for AR-276. No bacterial killing was observed for MIN at any concentration against AR-279. AMP/SUL was not bactericidal at any concentration against any of the four isolates, and RIF was only bactericidal against AR-276 (2 × and 4 × MIC). All isolates grew above the starting inoculum by 24 h in the presence of each antibiotic at 0.5 × MIC (the concentrations also used in combinations).

The combination of PMB and MER was bactericidal and synergistic against AR-276, AR-279, and AR-285, with a mean reduction of 5.11 log_10_ CFU/mL. In contrast, no bacterial killing was noted for PMB/MER against AR-313. PMB/MIN only displayed synergy against isolate AR-313 (2.28 log_10_ CFU/mL reduction) while the other isolates grew above the starting inoculum by 24 h in the presence of this combination. The combination of PMB and AMP/SUL was bactericidal against isolates AR-276, AR-285, and AR-313 at 24 h and synergy was observed against all four isolates. PMB and RIF were bactericidal and synergistic against AR-279 and AR-313. AR-285 regrew between 8 and 24 h in the presence of this combination.

### 3.3. Biofilm Time-Kill Assays

**Figure 2:**
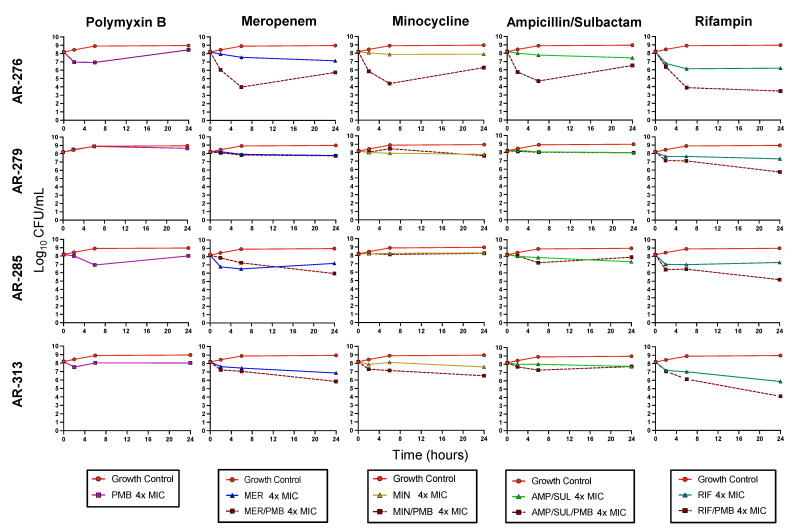
Mean viable bacterial counts of CRAB biofilms in static time-kill experiment. For combinations, each antibiotic was tested at a concentration of 4 × MIC. PMB, polymyxin B; MER, meropenem; MIN, minocycline; AMP/SUL, ampicillin/sulbactam; RIF, rifampin.

None of the antibiotics were bactericidal against any of the CRAB isolates as monotherapy when grown as biofilms (Figure 2). However, each monotherapy had relatively consistent activity against the four CRAB isolates. PMB, MER, MIN, and AMP/SUL caused mean reductions (±SD) across all isolates of −0.10 ± 0.32 (− denotes growth above baseline), 0.94 ± 0.37, 0.30 ± 0.31, and 0.86 ± 0.79, log_10_ CFU/mL at 24 h, respectively. RIF was the most active monotherapy against the biofilms at 4 × MIC with 2.32 ± 0.75 log_10_ CFU/mL mean reductions against the four isolates.

PMB and RIF was the most active combination against the biofilms. This combination was bactericidal against AR-276 and AR-313 and caused a 2.99 log_10_ CFU/mL reduction against AR-285. The combination of PMB and RIF was also synergistic against AR-276 and AR-285. The mean bacterial reduction against all four isolates caused by PMB combined with RIF was 3.55 log_10_ CFU/mL at 24 h. PMB/MER was not bactericidal or synergistic against any isolate at 24 h, but the combination was on average 0.92 log_10_ CFU/mL more active than the most active monotherapy. Similarly, combinations between PMB and MIN were 0.72 log_10_ CFU/mL more active than the most active monotherapy. The PMB/AMP/SUL combination only showed better killing activity than the monotherapies against AR-276. Each combination was the most active against AR-276 compared to the other isolates. Synergy was observed for all combinations at 6 h against this isolate, though only PMB/RIF was still synergistic at 24 h.

## 4. Discussion

The optimal approach to antibiotic therapy for infections caused by CRAB has not yet been clearly defined. Combinations containing PMB are one suggested treatment strategy, particularly for moderate to severe CRAB infections [7,27]. Although several studies have investigated polymyxin-based combinations for CRAB infections, very few have sought to identify the most active combinations against both planktonic and biofilm populations simultaneously. In the present study, we found that PMB combined with MER, AMP/SUL, or RIF was synergistic against planktonic populations of most of the tested CRAB isolates. However, only the combination of PMB and RIF retained bactericidal activity against any of the isolates when grown as biofilms. Optimizing combinations against both planktonic and biofilm CRAB populations may enhance the translation of in vitro studies to patients, where both phases of growth can exist in some types of infections.

In agreement with previous studies, our results demonstrate that CRAB biofilms generally exhibit increased antibiotic tolerance compared to planktonic cells [28]. Similar to the present study, one previous in vitro study also failed to show synergy or bactericidal activity between a polymyxin and MER against CRAB biofilms [25]. The accumulation of carbapenemases in the biofilm likely contributes to the difference in killing observed between planktonic and biofilm experiments for this combination [29]. Increased tolerance by biofilms to the combination of a polymyxin and MER may in part explain the inconsistent correlation between in vitro studies and clinical trials for CRAB infections, since the in vitro studies rarely considered both planktonic and biofilm populations. In vitro time-kill and checkerboard studies have primarily shown that polymyxins combined with carbapenems enhance the killing of CRAB compared to polymyxin monotherapy [30], although clinical studies have been less conclusive. The AIDA randomized open-label controlled trial compared colistin monotherapy (9-million-unit loading dose, followed by 4.5 million units every 12 h) to colistin plus MER (2 g prolonged infusion every 8 h) in patients with bacteremia, pneumonia or urosepsis and found no significant difference in clinical failure at day 14 between groups [4]. In this study, 77% of patients had infections caused by CRAB and nearly all of these isolates had MER MICs ≥64 mg/L. In a secondary analysis of the isolates from the AIDA study, Nutman et al. showed that there was no correlation between synergy detected by checkerboard studies (planktonic) and patient outcomes [31]. However, no consideration for activity against biofilms was made in this study. Taken together, these data do not support the use of a polymyxin plus MER for CRAB infections, which may for some infections be related to the combination’s diminished activity against biofilms.

Similar to the combination of PMB and MER, PMB plus AMP/SUL was highly active against planktonic cells but displayed minimal activity against biofilms. Wang et al. also did not observe synergy or bactericidal activity for the combination of colistin and SUL against CRAB biofilms [25]. A previous study conducted by Lenhard et al. showed that AMP/SUL can effectively eradicate CRAB in a hollow fiber infection model (planktonic) despite failing to induce bacterial killing against the same isolates in a time-kill assay (planktonic) [32]. Lenhard et al. propose that the activity of AMP/SUL observed within the HFIM, but not time-kill assays, was a result of the ability of CRAB to rapidly divide within the hollow fiber infection model. β-lactam activity is dependent on the rate of cell division. Indeed, the rate of replication, which is slower in biofilms, may also be a reason why neither of the combinations with a β-lactam (MER or AMP/SUL) were bactericidal or synergistic against CRAB biofilms [29]. Recent IDSA guidance suggests using combinations that include high-dose AMP/SUL with either MIN, tigecycline, or PMB as the preferred treatment approach for moderate to severe infections caused by CRAB [27]. One small randomized controlled trial (39 patients) compared colistin monotherapy (3 million units every 8 h) to colistin plus AMP/SUL (24 g of AMP/SUL per day) and found significantly higher rates of clinical improvement on day five by the combination, but the mortality rates at 28 days were not significantly different between groups. In addition to the small size, this study is also limited by the unblinded nature of the trial design and variability in antibiotic treatments after day five. Although AMP/SUL has displayed promising activity against planktonic CRAB populations in various studies, additional clinical trials are necessary to validate its efficacy. Alternative antibiotic regimens or other antibiotics in combination with AMP/SUL may be warranted to treat infections with biofilms.

PMB and RIF was the most consistently active combination against both populations of CRAB isolates in the present study. Several previous studies have assessed this combination against planktonically growing *A. baumannii* and together found synergy against >50% of clinical isolates [33]. In a mouse thigh-infection model, the combination of colistin and RIF was synergistic against all 12 CRAB isolates tested and was significantly more active than combinations with colistin plus either MER, tigecycline, fosfomycin, or SUL [34]. Although RIF has well-described antibiofilm effects against other pathogens such as *S. aureus* [35], only a few studies have assessed its activity against CRAB biofilms [36,37]. RIF has also been shown to suppress the amplification of polymyxin resistance in vitro [38,39]. A combination of colistin and RIF has also been compared to colistin monotherapy in patients. Durante-Mangoni et al. conducted a randomized open-label clinical trial comparing colistin monotherapy (2 MU IV every 8 h) to a combination of colistin plus RIF (600 mg IV every 12 h) for patients with life-threatening infections caused by CRAB [5]. They found no significant difference in 30-day all-cause mortality between treatment groups, and the authors suggested that RIF should not be considered for routine use in combination with colistin for CRAB infections. However, there was a significant increase in microbiologic eradication in patients who received the combination of colistin plus RIF. There were also several important limitations to this study that make it difficult to draw conclusions from, including the suboptimal colistin dose (i.e., no loading dose, and relatively low maintenance dose) and relatively low number of enrolled patients [7]. Thus, additional studies are warranted to test the combination of a polymyxin and RIF, especially for infections that contain both biofilm and planktonic populations.

There are several limitations to the current study. First, we only tested four CRAB isolates that can each form biofilms, which may limit the generalizability of our results. Future studies should expand testing to include additional clinical isolates. Second, we used static in vitro time-kill assays that do not mimic human drug PK. Additional studies that simulate dynamic antibiotic concentrations will be important to validate these findings. The planktonic time-kill assays were also conducted at larger volumes than the broth microdilution MICs, which may have led to an increased number of resistant subpopulations in the test-tubes at baseline (0 h). The presence of additional resistant subpopulations may in part explain why some antibiotic concentrations above the MIC failed to suppress growth. Nonetheless, these data add to the limited number of studies comparing the activity of polymyxin-based combinations between planktonic and biofilm populations of CRAB isolates.

## 5. Conclusions

PMB-based combinations with MER or AMP/SUL primarily had activity against planktonic populations of CRAB isolates. In contrast, PMB and RIF was the most active combination against both planktonic and biofilm populations of CRAB isolates. Thus, optimal antibiotic therapy may be dictated by the composition of the CRAB population at the site of infection (i.e., biofilm, planktonic, or both). The consistent activity of PMB and RIF against CRAB isolates in both phases of growth suggests that this combination may have utility for most infections, including those that harbor biofilms, such as ventilator-associated pneumonia or catheter-related bloodstream infections. Future in vitro and in vivo studies that include additional isolates are warranted to confirm these findings.

## Figures and Tables

**Table 1:** Genotypic characteristics of CRAB isolates and their MICs for antibiotics used in time-kill assays. Interpretations of MICs are provided in parentheses.

Isolate	Sequence Type	β-Lactamase *	MIC (mg/L)				
			Polymyxin B	Meropenem	Minocycline	Ampicillin/ Sulbactam	Rifampin
AR-276	ST530	*bla*_ADC-130_	1 (I)	8 (R)	0.125 (S)	8/4 (S)	8
AR-279	ST2	*bla*_TEM-1D_, ***bla*_OXA-23_**, ***bla*_OXA-66_**, *bla*_ADC-162_	0.5 (I)	64 (R)	4 (S)	64/32 (R)	32
AR-285	ST79	*bla*_TEM-1B_, ***bla*_OXA-65_**, *bla*_ADC-215_	0.5 (I)	256 (R)	0.125 (S)	32/16 (R)	4
AR-313	ST1	*bla*_TEM-1D_, ***bla*_OXA-23_**, ***bla*_OXA-69_**, *bla*_ADC-176_	0.5 (I)	16 (R)	4 (S)	64/32 (R)	4

MIC interpretations according to CLSI-ED32 (2022). * Bold β-Lactamase genes have been shown to hydrolyze carbapenems.

**Table 2:** Presence (+) or absence (−) of bactericidal activity and synergy for antibiotic combinations against planktonic and biofilm populations of CRAB isolates. Bactericidal activity was defined as a ≥3 log_10_ CFU/mL reduction in viable cell count at 24 h and synergy was defined as a ≥2 log_10_ CFU/mL reduction by the combination compared to the most active agent alone at 24 h. PMB, polymyxin B; MER, meropenem; MIN, minocycline; AMP/SUL, ampicillin/sulbactam; RIF, rifampin.

		PMB/MER		PMB/MIN		PMB/AMP/SUL		PMB/RIF	
		Bactericidal	Synergy	Bactericidal	Synergy	Bactericidal	Synergy	Bactericidal	Synergy
AR-276	Planktonic	+	+	−	−	+	+	−	+
	Biofilm	−	−	−	−	−	−	+	+
AR-279	Planktonic	+	+	−	−	−	+	+	+
	Biofilm	−	−	−	−	−	−	−	−
AR-285	Planktonic	+	+	−	−	+	+	−	−
	Biofilm	−	−	−	−	−	−	−	+
AR-313	Planktonic	−	−	−	+	+	+	+	+
	Biofilm	−	−	−	−	−	−	+	−

## Data Availability

Publicly available sequences were analyzed in this study. This data can be found through the following biosample accession numbers: SAMN04901666, SAMN04901669, SAMN04901675, and SAMN04901703.

## Supplementary Materials

The following are available online at https://www.mdpi.com/article/10.3390/antibiotics11070959/s1: Table S1. *A. baumannii* mean bacterial density (log_10_ CFU/mL ± SD) after 2, 6, and 24 h exposure to antibiotics in time-kill assays for both biofilm and planktonic populations.
