# Supplementary material for: Combatting Planktonic and Biofilm Populations of Carbapenem-Resistant Acinetobacter baumannii with Polymyxin-Based Combinations
Source: Antibiotics (Basel). 2022 Jul 16;11(7):959. doi: 10.3390/antibiotics11070959 (PMC9312021; doi:10.3390/antibiotics11070959)
Supplement: Supplementary file 1 [file antibiotics-11-00959-s001.zip › antibiotics-1732071-supplementary.pdf]

TABLE S1. *A. baumannii* mean bacterial density (log_{10} CFU/mL ± SD) after 2, 6, and 24 h exposure to antibiotics in time-kill assays for both biofilm and planktonic population.

		BIOFILM POPULATION [a]			PLANKTONIC POPULATION [b]		
**Isolate**	**Antibiotic**	**Bacterial Density (log_{10} CFU/mL) ± SD**					
		**2 h**	**6 h**	**24 h**	**2 h**	**6 h**	**24 h**
**AR-276**	PMB	6.97 ± 0.29	6.93 ± 0.76	8.45 ± 1.81	3.95 ± 0.02	4.06 ± 0.29	8.07 ± 0.08
	AMP/SUL	8.00 ± 0.05	7.77 ± 0.06	7.44 ± 0.14	6.29 ± 0.04	6.90 ± 0.16	8.43 ± 0.36
	MIN	8.04 ± 0.10	7.85 ± 0.60	7.89 ± 0.07	7.23 ± 0.06	7.60 ± 0.27	7.88 ± 0.98
	MER	7.95 ± 0.07	7.56 ± 0.10	7.14 ± 0.16	6.49 ± 0.08	5.92 ± 0.07	6.38 ± 0.05
	RIF	6.77 ± 0.15	6.13 ± 0.07	6.20 ± 0.43	5.24 ± 0.25	4.80 ± 1.35	8.25 ± 0.41
	PMB + AMP/SUL	5.75 ± 0.06	4.66 ± 0.69	6.53 ± 3.47	4.05 ± 0.09	2.70 ± 0.12	1.31 ± 1.13
	PMB + MIN	5.84 ± 0.43	4.37 ± 0.39	6.28 ± 3.02	4.21 ± 0.04	4.14 ± 0.21	6.93 ± 2.33
	PMB + MER	6.03 ± 0.54	3.99 ± 0.66	5.74 ± 2.81	4.41 ± 0.06	2.60 ± 0.00	0.00 ± 0.00
	PMB + RIF	6.36 ± 0.28	3.87 ± 0.83	3.46 ± 0.34	0.00 ± 0.00	0.00 ± 0.00	5.11 ± 3.83
**AR-279**	PMB	8.50 ± 0.07	8.88 ± 0.15	8.65 ± 0.01	5.46 ± 0.02	5.45 ± 0.64	7.97 ± 0.13
	AMP/SUL	8.22 ± 0.00	8.05 ± 0.00	7.97 ± 0.00	6.38 ± 0.05	7.11 ± 0.05	8.16 ± 0.08
	MIN	8.03 ± 0.01	7.93 ± 0.00	7.80 ± 0.00	7.48 ± 0.02	7.98 ± 0.01	8.07 ± 0.08
	MER	8.21 ± 0.00	7.89 ± 0.01	7.76 ± 0.01	5.74 ± 0.13	6.36 ± 0.01	8.27 ± 0.05
	RIF	7.64 ± 0.21	7.65 ± 0.10	7.36 ± 0.04	6.11 ± 0.05	5.79 ± 0.20	8.72 ± 0.28
	PMB + AMP/SUL	8.10 ± 0.02	8.01 ± 0.01	7.95 ± 0.01	5.46 ± 0.02	4.37 ± 0.01	4.29 ± 0.43
	PMB + MIN	8.06 ± 0.01	8.47 ± 0.39	7.63 ± 3.02	5.34 ± 0.17	4.60 ± 0.00	7.32 ± 0.29
	PMB + MER	8.06 ± 0.02	7.80 ± 0.02	7.70 ± 0.01	5.45 ± 0.23	4.27 ± 0.18	2.95 ± 0.64
	PMB + RIF	7.15 ± 0.08	7.10 ± 0.33	5.77 ± 0.32	4.19 ± 1.20	1.70 ± 1.41	0.00 ± 0.00
**AR-285**	PMB	8.00 ± 0.17	6.92 ± 0.35	8.01 ± 0.30	5.03 ± 0.01	5.01 ± 0.09	7.31 ± 0.18
	AMP/SUL	8.00 ± 0.49	7.85 ± 0.20	7.34 ± 0.16	6.03 ± 0.05	6.90 ± 0.05	7.75 ± 0.00
	MIN	8.20 ± 0.32	8.26 ± 0.17	8.30 ± 0.09	6.78 ± 0.02	7.94 ± 0.02	7.88 ± 0.23
	MER	6.77 ± 0.17	6.52 ± 0.06	7.18 ± 0.81	6.08 ± 0.03	5.46 ± 0.20	7.90 ± 0.02
	RIF	7.03 ± 0.25	7.00 ± 0.47	7.27 ± 1.16	6.93 ± 0.04	7.15 ± 0.55	8.19 ± 0.21
	PMB + AMP/SUL	8.05 ± 0.34	7.23 ± 0.18	7.88 ± 0.65	4.91 ± 0.08	2.46 ± 0.02	0.00 ± 0.00
	PMB + MIN	8.22 ± 0.18	8.12 ± 0.28	8.25 ± 0.45	4.75 ± 0.05	2.49 ± 0.02	7.19 ± 0.96
	PMB + MER	7.85 ± 0.06	7.24 ± 0.09	5.96 ± 1.07	4.83 ± 0.12	1.85 ± 0.28	0.00 ± 0.00
	PMB + RIF	6.40 ± 0.21	6.46 ± 0.23	5.19 ± 0.21	3.83 ± 0.71	1.85 ± 1.52	6.60 ± 1.10
**AR-313**	PMB	7.53 ± 0.03	8.03 ± 0.01	8.02 ± 0.17	5.36 ± 0.05	5.03 ± 0.03	7.31 ± 0.98
	AMP/SUL	7.99 ± 0.20	8.00 ± 0.13	7.74 ± 0.09	6.38 ± 0.08	7.04 ± 0.30	8.20 ± 0.12
	MIN	7.86 ± 0.25	8.11 ± 0.16	7.55 ± 0.24	6.05 ± 0.02	5.32 ± 0.08	7.77 ± 0.14
	MER	7.63 ± 0.00	7.46 ± 0.11	6.87 ± 0.08	6.51 ± 0.04	7.85 ± 0.01	8.57 ± 0.08
	RIF	7.18 ± 0.10	7.02 ± 0.03	5.86 ± 0.73	5.64 ± 0.08	6.26 ± 0.82	8.89 ± 0.14
	PMB + AMP/SUL	7.70 ± 0.04	7.30 ± 0.12	7.73 ± 0.76	5.00 ± 0.27	4.16 ± 0.54	1.31 ± 1.33
	PMB + MIN	7.29 ± 0.18	7.13 ± 0.11	6.51 ± 0.51	5.20 ± 0.08	4.27 ± 0.06	3.85 ± 2.94
	PMB + MER	7.23 ± 0.14	7.07 ± 0.20	5.87 ± 0.42	5.32 ± 0.03	4.01 ± 0.07	6.39 ± 3.11
	PMB + RIF	7.07 ± 0.03	6.14 ± 0.27	4.08 ± 0.23	4.16 ± 0.16	0.00 ± 0.00	0.00 ± 0.00

PMB, polymyxin B; AMP/SUL, ampicillin/sulbactam; MIN, minocycline; MER, meropenem; RIF, rifampin

[a] Monotherapies and combinations were performed at 4x MIC against biofilms at a starting inoculum of ~10^8 CFU/mL

[b] Monotherapies and combinations were performed at 0.5x MIC against planktonic cells at a starting inoculum of ~10^6 CFU/mL
